# Effects of Obstacles on the Dynamics of Kinesins, Including Velocity and Run Length, Predicted by a Model of Two Dimensional Motion

**DOI:** 10.1371/journal.pone.0147676

**Published:** 2016-01-25

**Authors:** Woochul Nam, Bogdan I. Epureanu

**Affiliations:** University of Michigan, Ann Arbor, Michigan 48109-2125, United States of America; A*STAR Bioinformatics Institute, SINGAPORE

## Abstract

Kinesins are molecular motors which walk along microtubules by moving their heads to different binding sites. The motion of kinesin is realized by a conformational change in the structure of the kinesin molecule and by a diffusion of one of its two heads. In this study, a novel model is developed to account for the 2D diffusion of kinesin heads to several neighboring binding sites (near the surface of microtubules). To determine the direction of the next step of a kinesin molecule, this model considers the extension in the neck linkers of kinesin and the dynamic behavior of the coiled-coil structure of the kinesin neck. Also, the mechanical interference between kinesins and obstacles anchored on the microtubules is characterized. The model predicts that both the kinesin velocity and run length (i.e., the walking distance before detaching from the microtubule) are reduced by static obstacles. The run length is decreased more significantly by static obstacles than the velocity. Moreover, our model is able to predict the motion of kinesin when other (several) motors also move along the same microtubule. Furthermore, it suggests that the effect of mechanical interaction/interference between motors is much weaker than the effect of static obstacles. Our newly developed model can be used to address unanswered questions regarding degraded transport caused by the presence of excessive tau proteins on microtubules.

## Introduction

Cells use various motor proteins for active transport along the cytoskeleton. Among these proteins, kinesin-1 is responsible for the anterograde transport along microtubules (MTs). The two identical heads of kinesin-1 are connected to their neck linkers (NLs), which are composed of fourteen amino acids (AAs). NLs are wrapped around each other at the neck forming a coiled-coil structure. The neck is attached to a cargo linker, whose other end is connected to a tail domain where a cargo binds. MTs consist of numerous tubulins where the kinesin heads can successively bind in a stepping process. Kinesins repeat one mechanochemical cycle per step. At every cycle, one head moves to a different binding site approximately 16 nm away from the previous one. Thus, the cargo attached to the kinesin moves approximately 8 nm per step. The time required to complete one mechanochemical cycle is determined by the interaction of kinesin heads and adenosine triphosphate (ATP) [1–6]. The average period of the step is about 10 ms at high ATP concentrations. Hence, the velocity of kinesins is about 800 nm/s, and varies with the load acting on the cargo [4, 7–9]. Kinesins have a run length of about 1 *μ*m, i.e., they can move about 1 *μ*m before they are released from the MTs [10–14].

These characteristics of kinesins can be changed by other proteins bound on the MTs. Recent experiments suggest that other proteins attached to the MT surface can act as obstacles for kinesins [15–21]. Kinesin motion can be affected also by other nearby kinesins. Leduc et al. [15] observed that the velocity of kinesins decreases dramatically when the number of motors on a MT exceeds a critical value. Furthermore, nonmotile kinesin mutants bound on MTs decrease the velocity and the run length of other walking kinesins [17]. Moreover, the motion of kinesin is inhibited also by MT associated proteins such as tau. The effects of tau proteins on kinesin and dynein have been compared by Dixit et al. [18], where it was revealed that the dependency of kinesins on tau proteins bound to the MT is much stronger than that of dynein. Experiments with cargoes inside cells also suggest that tau proteins decrease the transport distance of molecular motors [20].

Several models have been proposed to describe the motion of kinesin heads [22–27], focusing mainly on the one dimensional motion of the kinesin head. However, several experiments with static obstacles suggest that kinesins can bypass obstacles by executing 2D movements along the MT surface [17, 19, 28]. To capture this 2D stepping motion, we first develop a new model to capture the diffusive motion of kinesin heads in the absence of obstacles. Various equations (i.e., Fokker-Plank equation), models (i.e., a worm-like chain model (WLC) [29]), and experimental results (i.e., unfolding of the coiled-coil structure [30]) are coupled in our model. Next, a deterministic model and stochastic model are developed by using the diffusion model to predict the motion of kinesin in the presence of obstacles.

## Model

**Fig 1:**
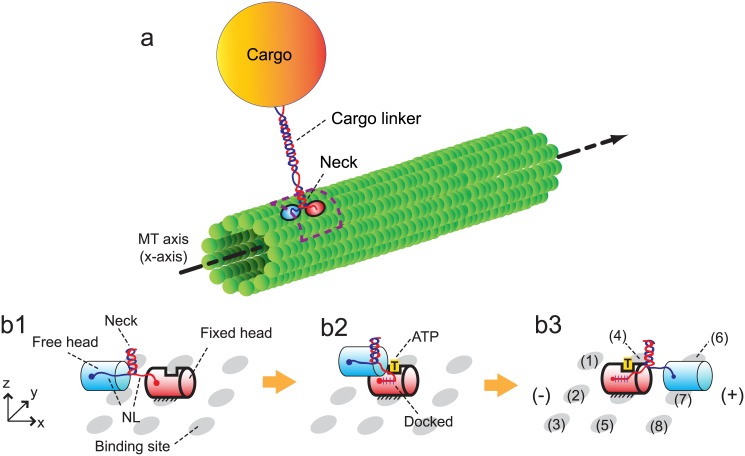
Walking motion of a kinesin molecule on the MT. (a) depicts one kinesin molecule transporting a cargo by walking on the MT. The dotted box indicates the domain where the motion of the kinesin head is realized. (b) shows the components of the kinesin structure and the procedure for its walking motion. The kinesin has one pair of identical heads and NLs. *x* represents the direction of MT-axis, and *y* is the tangential direction of the MT. *xy*-plane represents the outer surface of the MT. The cylinder with bold outlines denotes the fixed head on the MT, and the cylinder with thin outlines is the free head. The neighboring binding sites of the kinesin are shown as gray ellipses. The plus and minus signs denote the polarity of the MT.

The walk cycle of kinesins is realized by their structural changes and by diffusion of its heads. It begins from a state where ATP is not bound on the fixed head of the kinesin, as described in Fig 1b1. The structural change from Fig 1b1 to 1b2 is caused by the binding of ATP to the fixed head. This binding creates the bonds between the fixed head and its NL. After the structural change, the free head diffuses until it reaches one of the eight neighboring binding sites (1)–(8), as shown in Fig 1b3. The diffusive motion of the free head is important because it determines the direction of the kinesin walk.

The motion caused by the structural changes is considered in our study by using the results of a previous study [31]. Specifically, the head which is not bound to the MT moves about 6 nm along the MT axis in a very short time immediately following a structural change. The subsequent motion (i.e., diffusion of the free head) is captured by using the developed methods explained below.

### Forces acting on kinesin structure

**Fig 2:**
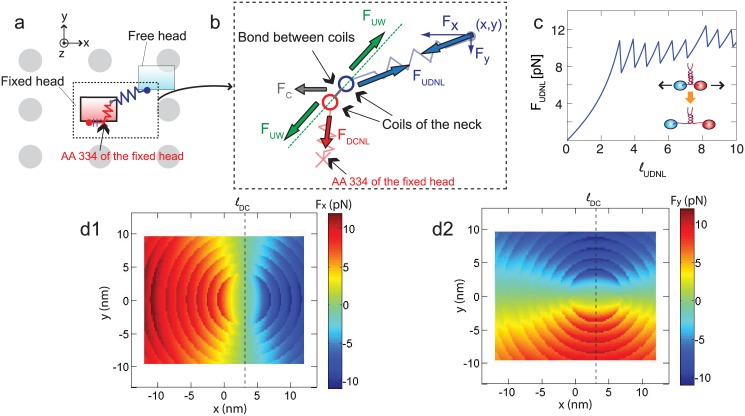
Spring model of kinesin NLs. (a) shows a kinesin molecule and the binding sites around it. Note that the directions of *x*, *y*, and *z* axis are the same with those in Fig 1, and the view direction is along the *z* axis. NLs are depicted with springs, and gray circles represent the neighboring binding sites. (b) is a schematic cross section through the coiled-coil structure of the neck. The coils are bonded to each other by using the interactions of their AA residues. The neck is connected to two springs corresponding to the docked NL and undocked NL. (c) shows the changes of the force (*F*_UDNL_) over the length (*ℓ*_UDNL_) of undocked NL. (d1) depicts the calculated *F*_*x*_(*x*, *y*), where (*x*, *y*) denotes the *x* and *y* positions of the free head. (d2) shows the calculated *F*_*y*_(*x*, *y*).

Forces acting on kinesin structure need to be calculated in order to predict the diffusive motion of the free head. Because the free head is connected to the fixed head through two NLs which are nonlinear springs, as shown in Fig 2a, the springs have to be stretched for the free head to reach the binding sites.

The forces acting on the free head (i.e., *F*_*x*_ and *F*_*y*_ in Fig 2b) are calculated by considering three different forces: forces, *F*_DCNL_, acting on the docked NL, forces, *F*_UDNL_, acting on the undocked NL, and forces, *F*_c_, transferred from the cargo to the neck via the cargo linker. For a given position of the free head, the neck is located at the position where the vector summation of these forces is zero. *F*_DCNL_ and *F*_UDNL_ are calculated by using the WLC [29, 32] as

$$\begin{array}{c} F_{\text{NL}}=\frac{k_{B}T}{\ell_{p}}\left(\frac{1}{4}{\left(1-\frac{\ell_{\text{NL}}}{\ell_{c}}\right)}^{-2}+\frac{\ell_{\text{NL}}}{\ell_{c}}-\frac{1}{4}\right), \end{array}$$ (1)

where *k*_*B*_ is Boltzmann constant, *T* (= 300 K) is the absolute temperature, and ℓ_*p*_ (= 0.6 nm) is the persistence length. ℓ_*c*_ is the contour length of the NLs which can be calculated as *N*_*AA*_ ℓ_*a*_, where ℓ_*a*_ (= 0.4 nm) is the contour length between two adjacent AAs. *N*_*AA*_ denotes the number of AAs in the NLs. *N*_*AA*_ is equal to *N*_*AA*, 0_ + 3.5 *n*_uw_, where *N*_*AA*, 0_ is the number of AAs in the NLs when every bond in the neck is intact. *n*_uw_ is the number of unwound turns of the neck. Because the unfolding of one turn increases the number of AAs by 3 or 4 for each NL, the coefficient 3.5 is used when calculating *N*_*AA*_ with *n*_uw_. *N*_*AA*, 0_ is 14 (AA 325–338) for the undocked NL and 4 (AA 335–338) for the docked NL because AA 334 of the docked NL is tightly bound to the fixed head with two backbone hydrogen bonds [33]. The neck consists of two coils which are connected to each NL, as shown in Fig 1. The coils are coiled with each other for about 10 turns, and that coiled-coil structure is maintained by hydrophobic interactions [34]. In experiments performed by Bornschlogl et al. [30], it is observed that each turn of the neck can be unwound when stretching forces of about 10 pN are applied. This behavior changes the relation between the force acted in the NL and its length when the force reaches the unwinding force values, as shown in Fig 2c. The force is also affected by ℓ_*DC*_ which is the distance between AA 324 and AA 334 of the NL in a condition when they are docked to the fixed head. This length determines the position of AA 334 of the docked NL, as shown in Fig 2a. Thus, the magnitude of the force in the x and y directions (i.e., *F*_*x*_ and *F*_*y*_) acting on the free head is symmetric about *x* = ℓ_*DC*_, as shown in Fig 2d. The refolding of the neck is also considered by assuming that the force-displacement relation for stretching is the same when releasing that stretching.

When calculating *F*_*x*_ and *F*_*y*_, the state is first considered where every chemical bond between two coils of the neck is connected (i.e., *n*_uw_ = 0). The moment caused by *F*_c_ on the neck is negligible because *F*_c_ acts at the center of the neck. The two coils align along a line which is indicated with the dotted line in Fig 2b, so that the net moment caused by *F*_UDNL_ and *F*_DCNL_ is zero. However, both coils are stretched by the force *F*_UW_ along the dotted line in Fig 2b. The magnitude of *F*_UW_ can be calculated using *F*_c_, *F*_DNL_, and *F*_UDNL_. The bonds of the neck can be broken or intact depending on *F*_UW_. If *F*_UW_ is less than the force required to unwind the first bond of the coiled-coil structure, then *F*_*x*_ and *F*_*y*_ are determined by *F*_UDNL_. If the calculated *F*_UW_ is larger than the force to unwind the first bond, the simulation step is performed again, unwinding the first turn of the neck (i.e., *n*_uw_ = 1). This procedure is repeated until *F*_UW_ becomes less than the force required to unwind the subsequent turn.

### Diffusive motion of kinesin head

To determine the direction of the kinesin step, the diffusive motion of the free head has to be considered. However, calculating that diffusive motion at every walk cycle requires intensive computations. To reduce computation effort dramatically, the following method is used. First, the probability of the direction of step is calculated by solving the Fokker-Plank equation on the position of the free head. Then, a random number is generated at every step of kinesins. Next, the direction of each step is determined by comparing the random number to the calculated probability. Because a solution of the Fokker-Plank equation can be used at every step of kinesins, this method enables us to determine the stochastic two dimensional walking motion of kinesin with higher computational efficiency.

The probability density for the position of the free head is calculated over time by solving the following Fokker-Plank equation (in a domain depicted in Fig 1a with a dotted box)

$$\begin{array}{c} \frac{\partial p}{\partial t}=D\frac{\partial^{2}p}{\partial x^{2}}+D\frac{\partial^{2}p}{\partial y^{2}}-\frac{\partial }{\partial x}\left(\frac{F_{x}}{\gamma }p\right)-\frac{\partial }{\partial y}\left(\frac{F_{y}}{\gamma }p\right), \end{array}$$ (2)

where *x* and *y* are coordinates of the free head with respect to the fixed head along the MT-axis and along the tangential direction of the MT, respectively. The software COMSOL is used to solve this partial differential equation with the finite element method. *p* is the probability density function of the position of the free head. *D* is the diffusion coefficient, and *γ* is the drag coefficient of the free head in water. The head is assumed cylindrical with a length of 7 nm and a diameter of 4.5 nm. These dimensions of the head are obtained from a previous study on the crystal structure of kinesin heads [35]. *γ* is calculated to be 5.625 × 10^−8^ g/s by using the method of Swanson et al. [36] for the drag coefficient for a cylinder moving at low Reynolds numbers. Then, the diffusion coefficient can be calculated as 73.6 *μ*m^2^/s for a temperature of 300 K. Note that the dependency of *γ* and *D* on the direction of motion is negligible for such cylinder dimensions [36]. The effect of the motion along the radial direction of the MT is assumed to be negligible.

**Fig 3:**
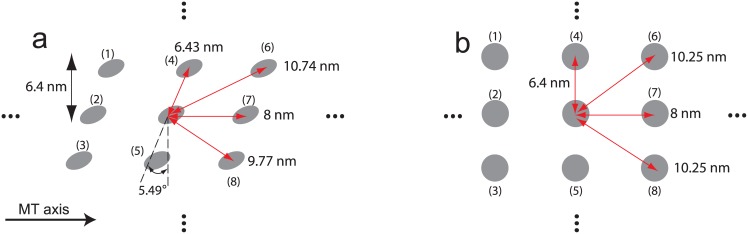
Lattice array of MTs and the distance between adjacent binding sites. (a) shows the binding sites for MTs of a helical structure. (b) depicts the binding sites in the absence of a mismatch in the lattice array.

The probability of stepping to the left sites (i.e., site (4) or (6) in Fig 3) can be different to the probability of walking to the right sites (i.e., site (5) or (8) in Fig 3) because there is a mismatch in the lattice of MTs due to a variable number of protofilaments. However, Yildiz et al. [37] observed that the probability of kinesin to move left is the same as the probability to move right. This observation suggests that the effects of the mismatch in the lattice of MTs are not significant. To confirm that this effect is small, the distance from the binding sites to the center of the fixed head is calculated. For a MT diameter of 26.5 nm and a number of 13 protofilaments, the angle of the mismatch is approximately 5.49°(= atan(8/(26.5 × *π*))). For a helical MT structure, the distances to the two diagonal sites (left and right) are 10.74 nm and 9.77 nm, respectively, as shown in Fig 3a. In the absence of a mismatch in the lattice array, these distances are both 10.25 nm. Because the change in the distance due to a lattice mismatch is not considerable (i.e., only about 5%), isotropic diffusivity can be used in this study.

**Fig 4:**
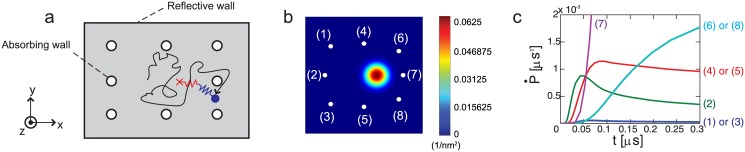
Diffusive motion of kinesin head in the absence of obstacles. The gray area of (a) describes the domain where Eq 2 is solved. (b) is the spatial probability density of the position of the free head after 1 *μ*s after the diffusion has started. (c) shows the rate of the probability density flow out through each absorbing boundary.

Two types of boundaries are considered, as shown in Fig 4a. Absorbing boundary conditions (i.e., *p* = 0) are applied at the binding sites. Reflective boundary conditions (i.e., no flux along the normal direction to the boundary) are used for the other boundaries.

Fig 4b shows one example of the spatial probability density for the free head. The probability that the free head binds to a certain binding site is obtained by integrating over time the flux $\dot{P}$ of the probability flowing out through the boundaries of the site (which is shown in Fig 4c).

### Binding with tilted posture

**Fig 5:**
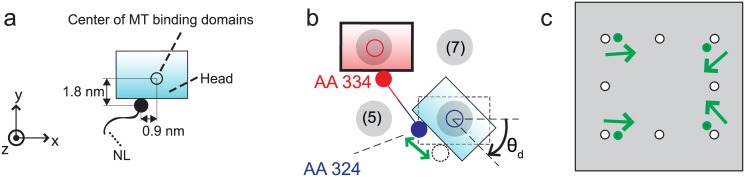
Free head when binding to diagonal binding sites. (a) depicts the positions of AA 324 with a filled circle. The geometric center of the binding domains which interact with binding sites is shown as a hollow circle. (b) depicts the assumption that the free head is likely to bind to the diagonal site with a tilted posture. (c) shows with filled circles the position of the absorbing boundaries of the diagonal sites which correspond to binding with a tilted posture.

To consider the effects of the affinity of the kinesin head to the binding sites, the positions of the specific AAs of the free head can be examined. First, AA 142–145, AA 273–281, AA 238 and AA 255 are responsible for binding on the MT [33]. Thus, it is assumed that the head binds to a binding site when the geometric center of these AAs reaches the binding site. Second, AA 324, which connects the NL to the head, is located about 0.9 nm away from that geometric center along the x direction and about 1.8 nm away along the y direction, as shown in Fig 5a. Due to this structure, when the free head is located at a diagonal site with a tilted posture, the length of the NLs to reach that site is shorter than the length when the free head reach the site with no rotation, as shown in Fig 5b. Thus, it is assumed that the binding to the diagonal site with tilted posture is favorable to the kinesin free head. The parameter *θ*_*d*_ represents the allowable tilting angle. We further hypothesize that if the free head is tilted with an angle larger than *θ*_*d*_, then the affinity between the binding site and the AAs of the free head which interacts with the binding sites is not strong enough to cause binding. When the geometric center of AAs responsible for binding is located at the diagonal binding site, and the tilted angle is less than *θ*_*d*_, the interaction between the head and the site is strong. Then, the head binds to the site. Subsequently, the head is aligned along the MT by that interaction. To mathematically model this behavior, the positions of the absorbing boundaries in the domain are calculated as follows. First, we locate the geometric center of the AAs responsible for binding of the free head at a certain binding site (e.g., site (8)), as shown in Fig 5b. Then, we rotate the free head with respect to the geometric center by an angle *θ*_*d*_. Then, the position of the binding is determined as the relative position of AA 324 of the free head with respect to the position of AA 334 of the fixed head. For example, when *θ*_*d*_ is 47°, the distances between the diagonal site and the fixed head decreases by 14.7% compared to the same distance when *θ*_*d*_ is 0°. Fig 5c shows the position of binding sites obtained using this method. Note that this behavior is only applicable to the diagonal sites because there is not enough space for the free head to tilt when the head is near the side, forward, or backward binding sites due to the geometric interference between the free head and the fixed head.

### Comparison to experimental data

**Fig 6:**
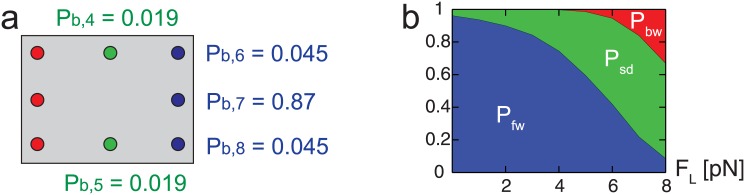
Probability of binding at neighboring sites. The numbers in (a) denote the probability to bind (*P*_*b*, *i*_) at each site in the absence of external loads. The probability *P*_*b*_ of backward steps is not indicated because its value is very small. (b) shows the changes in the probabilities of forward (*P*_fw_ = *P*_*b*, 6_ + *P*_*b*, 7_ + *P*_*b*, 8_), sideway (*P*_sd_ = *P*_*b*, 4_ + *P*_*b*, 5_), and backward (*P*_bw_ = *P*_*b*, 1_ + *P*_*b*, 2_ + *P*_*b*, 3_) steps over the external load.

The diffusion model has three parameters; ℓ_*DC*_, *θ*_*d*_, and *d*_side_, where *d*_side_ is the distance between two adjacent binding sites along the tangential direction of the MT. The values of these three parameters are determined by calculating the probability (*P*_*b*, *i*_) of the free head to bind at each neighboring binding site and by comparing it with experimental data. Yildiz et al. [37] tracked the motion of kinesin heads by labeling them with quantum dots. Their results show that 13% of the kinesin steps involve motion along the tangential direction of the MT, and 70% of that lateral motion occurs together with the motion toward the plus end of the MT. Also, the measurements of Nishiyama et al. [38] show that the probability of backward steps increases exponentially over the resisting load acting on the cargo. According to their observations, the probability ratio of forward and backward steps is about 1.3 when the resisting load is about 7 pN. A set of parameters (ℓ_*DC*_ = 2.9 nm, *d*_side_ = 6.4 nm, and *θ*_*d*_ = 47°) satisfies the probability of step observed in both experiments. The probabilities of forward, backward and sideway step in the absence of resisting loads are presented in Fig 6a. ℓ_*DC*_ and *d*_side_ are similar to the measured values of the previous studies. The value of ℓ_*DC*_ in the crystal structure of kinesin [39] is about 3 nm. Also, *d*_side_ of 6.4 nm can be obtained when the diameter of the MT is 26.5 nm. This diameter value is in the range of the actual diameters of MTs observed in the cell [40, 41].

### Effects of load on the probability of step

The external load acting on the cargo affects the probabilities of kinesin step. When an external load is applied to the cargo, it is transferred to the cargo linker. That force, *F*_*c*_, changes the force acting on the free head (i.e., *F*_*x*_ and *F*_*y*_). As a result, the probabilities of sideway and backward steps increase over the loads, as shown in Fig 6b. Note that the direction of the load is toward the minus end of the MT.

### Effects of unwinding the neck and binding with a tilted posture

Both the unwinding of the neck and the binding with tilted posture are necessary to obtain the probability of the direction of a step measured in experiments [37]. To check the effects of unwinding the neck on the diffusion, *P*_*b*, *i*_ is obtained using the model which allows the binding with tilted posture but does not allow neck unwinding. U_off_ + T_on_ in Table 1 represents this case. The equation used to calculate *F*_NL_ is the same as Eq 1 except that the value of *N*_*AA*_ is fixed as *N*_*AA*, 0_. Similarly, U_on_ + T_off_ represents the case of constraints which do not allow unwinding of the neck but allow binding with tilted posture. U_on_ + T_on_ represents the case where both unwinding of the neck and binding with a tilted posture are allowed. The binding probability presented in Table 1 suggests that both behaviors are necessary to obtain values similar to those *P*_*b*, *i*_ measured experimentally [37].

**Table 1:** Binding probability (*P*_*b*, *i*_) to each site for the model with various constraints.

Site	Experiment	U_on_ + T_on_	U_off_ + T_on_	U_on_ + T_off_
(4) or (5)	0.0195	0.0193	7 × 10^−5^	0.021
(6) or (8)	0.0455	0.0452	1.2 × 10^−6^	0.00169
(7)	0.869	0.871	0.99	0.954

### Number of binding sites occupied by kinesin

**Fig 7:**
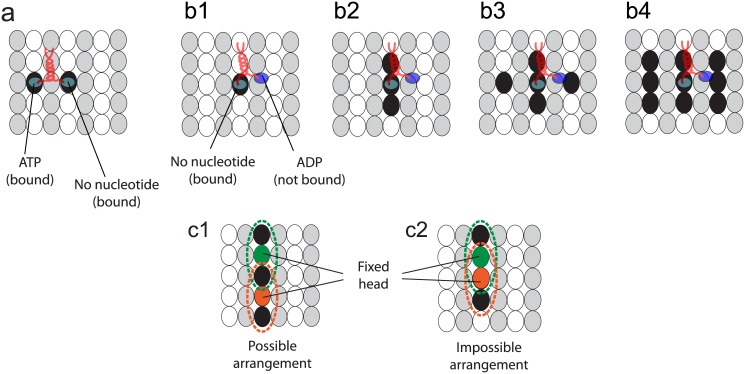
Binding sites occupied by kinesin heads. The black ellipses represent the binding sites occupied by the kinesin. The gray and white ellipses are *α* and *β* tubulins of the MT. Note that the kinesin head can only bind to *β* tubulins. (a1) Two sites are occupied by the kinesin when its two heads are strongly bound. (b) depicts possible scenarios when one of the kinesin heads can be unbound and the other head is strongly bound. The kinesin can occupy one (b1), three (b2), five (b3), or nine (b4) binding sites. (c1)-(c2) shows two examples of cases where sites occupied by two kinesin molecules are adjacent, allowing for interference. Both kinesins have one unbound head and one bound head. The dotted circles represent the sites occupied by kinesin heads.

The number of binding sites occupied by kinesin molecules depends on the chemical state of its head. The kinesin head has a strong affinity to the MT when it has ATP or no nucleotide [42]. The head has a low affinity to the MT if it has adenosine diphosphate (ADP). When one head has ATP and the other head has no nucleotide, as described in Fig 1b3, both heads are strongly bound to the MT. Thus, two sites are occupied by the kinesin in this state, as shown in Fig 7a.

When one head has no nucleotides or ATP and the other has ADP, as shown in Fig 1b1 or 1b2, one head diffuses around the (other) head which is strongly bound to the MT [43, 44]. For these states, the strongly bound head (i.e., the head with no nucleotide or with ATP) occupies a single site. Because the free head is diffusing around the fixed head, it is possible that the heads of other kinesin molecules cannot bind to the sites around this kinesin molecule. Several possible scenarios of binding sites, which are not accessible for other kinesin molecules, are studied, as shown in Fig 7b. Among them, the second case, depicted in Fig 7b2, is selected based on experiments performed by Telley et al. [17]. They observed the kinesin motion on MTs on which other immobile kinesins are attached. The state of the immobile kinesins is same with that of the walking kinesin when one of its head is bound to the MT and the other head is free. Also, the motion of walking kinesins depends on the number of binding sites occupied by the immobile kinesins. Thus, the velocity of walking kinesins in the presence of immobile kinesins can be used to determine the number of binding sites occupied by the kinesin in this state. Details are provided in the results.

Since the unbound heads do not remain at one site, they can share the same site at different times, as shown in Fig 7c1. However, a head cannot bind to a site if the site is affected by the unbound head of other kinesins, as shown in Fig 7c2.

## Results

The motion of kinesin is affected by the interaction between obstacles and kinesins and by the number of the obstacles on the MT. Our model is used to characterize the interaction by locating obstacles near a kinesin molecule. By using experiments, where the velocity and run length of kinesins are observed in the presence of immobile kinesins, the value of parameters regarding the interaction between kinesin molecules are obtained. Then, the velocity and run length of kinesins are calculated over the number of obstacles. Furthermore, the characterization of interaction between kinesins and obstacles enables us to predict the motion of kinesin when several types of molecular motors move along the same MT. In addition, the effects of the length of NLs and the probability of large steps (i.e. two binding sites away from the fixed head) are studied.

### Motion of kinesin in front of a single obstacle

**Fig 8:**
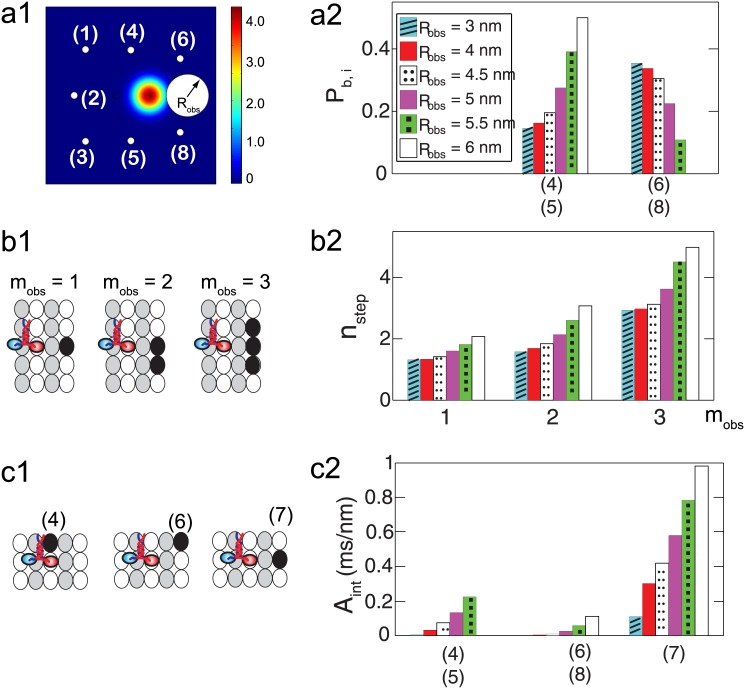
Motion of kinesin in front of a single obstacle. (a1) shows the probability density over the domain after 1 *μ*_s_ if a single obstacle of *m*_obs_ = 1 is ahead of the kinesin. The blocked region formed by the obstacle is included into the domain with the reflective boundary. (a2) denotes the probability to bind to each site. The numbers in parentheses represent the position of the sites shown in (a1). (b1) demonstrates different types of obstacles which occupy one, two or three binding sites. The black ellipses represent the binding sites occupied by a single obstacle. (b2) shows the number of kinesin steps (*n*_step_) to proceed 8 nm along the MT axis for various *m*_obs_. (c1) illustrates cases where one of the neighboring sites becomes unaccessible due to an obstacle. (c2) denotes the degree of interference for the cases shown in (c1).

To characterize the interaction between kinesins and their neighboring obstacles, the size of the region which the kinesin head cannot reach due to the obstacle (*R*_obs_), the number of binding sites occupied by a single obstacle (*m*_obs_), and the degree of interference (*A*_int_) between the kinesin and the obstacle are considered. When a binding site is occupied by obstacles, the free head is not able to reach it. Thus, a reflective boundary is located at that binding site. The shape of the reflective boundary is assumed to be circular, as shown in Fig 8a1. Next, the degree of interference (*A*_int_) between obstacles and the free head is calculated by using the probability density of the free head to reach the boundary as

$$\begin{array}{c} A_{\text{int}}=\int_{t_{0}}^{t_{f}}\oint_{C_{\text{obs}}}p\;d\ell \;dt, \end{array}$$ (3)

where *t*_0_ and *t*_*f*_ are the initial and final time, respectively, when solving Eq 2. *C*_obs_ is the reflective boundary of obstacles located near the kinesin. Then, the unbinding probability of kinesin per step can be calculated by using *A*_int_ as

$$\begin{array}{l} P_{\text{ub}}=P_{\text{ub}}^{0}+\Delta P_{\text{ub},\text{obs}},\\ \text{where }\Delta P_{\text{ub, obs}}=\alpha_{\text{obs}}A_{\text{int}}, \end{array}$$ (4)

where $P_{ub}^{0}$ is the unbinding probability per step when every neighboring binding site is not occupied by obstacles. $P_{ub}^{0}$ are obtained from our previous model [45]. Δ*P*_ub, obs_ is the change in the unbinding probability due to obstacles. Note that the kinesin dissociates from the MT mostly when one of its heads is diffusing [10, 45]. Thus, it is assumed that the increase of the unbinding probability due to obstacles during that diffusion is also very large compared to Δ*P*_ub, obs_ for other kinesin states. *α*_obs_ is the parameter of the model which calculates the probability to unbind when the free head interacts with the obstacles.

If the site ahead of the fixed head is occupied by a the small obstacle, the kinesin is likely to bypass it by moving forward to a diagonal site. However, for a large obstacle, the probability to move to one of the the side sites is larger than that of moving to a forward diagonal site, as shown in Fig 8a2. Thus, the kinesin needs a larger number of steps (*n*_step_) to bypass an obstacle when *R*_obs_ is large, as shown in Fig 8b2. *n*_step_ also increases with *m*_obs_ exponentially. Note that *A*_int_ is maximum when the forward site is occupied by an obstacle, as shown in Fig 8c2. This means that the interference of obstacles is most significant when they are located in front of kinesins.

### Static obstacles

The parameters describing the effects of obstacles (i.e., *R*_obs_, *m*_obs_, and *α*_obs_) depend on the type of obstacle. In this study, immobile kinesins which act as static obstacles for walking kinesins are characterized by using previously reported experimental data [17]. In those experiments, the number of obstacles (i.e., immobile kinesins) attached to the MT was 8% of the maximum number of obstacles (obtained when the MT is fully coated with the obstacles). The velocity and run length were observed to decrease to 81% and 57% compared to their values in the absence of obstacles. The values of *R*_obs_ and *m*_obs_ are obtained by using the changes in the kinesin velocity. The observed decrease of the run length is used to calculate the value of *α*_obs_.

#### Velocity in the presence of static obstacles

The motion of the heads of the immobile kinesins is the same with that of the walking kinesin when one of its head is bound to the MT and the other head is free. Thus, the binding site occupied by a single immobile kinesins of *m*_obs_ = 1 corresponds to Fig 7b1. The occupied sites by immobile kinesins of *m*_obs_ = 3, 5, and 9 correspond to Fig 7b2, 7b3 and 7b4, respectively. The behavior of the unbound head, which can share binding sites with other kinesins, is also considered as described in Fig 7c1. To estimate the number of immobile kinesins present in the experiment, we randomly distributed them until the MT was fully coated with immobile kinesins. When the MT is saturated with immobile kinesins, the molar ratio *ρ* of the immobile kinesins and tubulin dimers is calculated as 0.4352 for *m*_obs_ = 3, 0.3572 for *m*_obs_ = 5, and 0.1843 for *m*_obs_ = 9. When we compared the velocity predicted by the model to the velocity observed in the experiments, the molar ratio *ρ* of 0.08 is used for *m*_obs_ = 1, 0.0346 (= 8% × 0.4352) is used for *m*_obs_ = 3, *ρ* of 0.0286 (= 8% × 0.3572) is used for *m*_obs_ = 5, and *ρ* of 0.0147 (= 8% × 0.1843) is used for *m*_obs_ = 9.

To obtain the value of aforementioned parameters, we developed a mathematical equation capable of calculating the (average) velocity and run length for various numbers of obstacles by using the behavior of kinesin in front of single obstacles, as explained previously. Specifically, velocities can be calculated as

$$\begin{array}{c} V\left(\rho \right)≃V_{0}\frac{1}{1+\sum_{k}a_{k}\rho^{k}}, \end{array}$$ (5)

where *V*(*ρ*) is the velocity for a molar ratio *ρ*. *V*_0_ is the velocity in the absence of obstacles. The derivation of this equation is provided in S1 File. The coefficient *a*_*k*_ is determined by *n*_step_, which is calculated using the diffusion model. Table 2 shows the velocities obtained for *R*_obs_ = 4 and 5 nm and *m*_obs_ = 1, 3, 5, and 9. The values of these parameters were determined as *R*_obs_ = 5 nm and *m*_obs_ = 3 for the immobile kinesin obstacles. Note that *m*_obs_ = 3 is also used for the walking kinesin when one of its head is fixed and the other is not bound.

**Table 2:** Kinesin velocity for various *m*_obs_ and *R*_obs_.

*m*_obs_	*R*_obs_ [nm]	*V*/*V*_0_
1	4	0.99
1	5	0.98
3	4	0.88
3	5	0.82
5	4	0.9
5	5	0.85
9	4	0.94
9	5	0.92

#### Run length in the presence of static obstacles

The run length of the deterministic model can be calculated as

$$\begin{array}{c} RL\left(\rho \right)=RL_{0}\frac{1}{1+\sum_{k}b_{k}\rho^{k}}\frac{V(\rho )}{V_{0}}, \end{array}$$ (6)

where *RL*(*ρ*) and *RL*_0_ are the run length in the presence and absence of obstacles, respectively. The values of *b*_*k*_ depend on *α*_obs_. The derivation of this equation is provided in S1 File.

To obtain precise values of the parameters, the time and spatial resolutions used in experiments were applied to our stochastic model. The mathematical models in Eqs 5 and 6 are based on the assumption that the time and spatial resolution is sufficiently small. However, a finite resolution is used in the experiments. Thus, the velocity and run length are obtained again by using a stochastic mathematical model with the resolution of the experiments. The stochastic model was developed by integrating the diffusion model with a previously developed model which describes the stochastic motion of the kinesin in the absence of obstacles [45]. Details on the stochastic model are described in S1 File. The stochastic model with *R*_obs_ = 5 nm and *m*_obs_ = 3 provides velocities similar to those obtained using Eq 5. The value of *α*_obs_ which leads to a good match with experimentally observed run lengths is of 0.044 nm/ms.

It is worthy to note the effect of obstacles on the run length and velocity of kinesin. The run length decreases as the unbinding probability is increased due to obstacles. The effect of unbinding probability on the velocity depends on the concentration of obstacles anchored on the MT. When the concentration of obstacles on the MT is small, the velocity does not change. However, if the number of molecules is considerable, both the size of obstacles and the degree of interference (*A*_int_) should be considered. If the size of obstacles (which is considered by using *R*_obs_) is small, the probability regarding the direction of step does not change significantly. Thus, the change in velocity is very small. However, if *R*_obs_ is large and *A*_int_ is small, the kinesins have more chances to walk sideways. In this case, the velocity can be reduced over the concentration of obstacles. If both *R*_obs_ and *A*_int_ are large, both the probability to walk sideway and the unbinding probability increase. As a result, the kinesin is likely to unbind from the MT near obstacles before walking to side binding sites. Thus, the decrease in the velocity is not considerable.

### Moving obstacles

**Fig 9:**
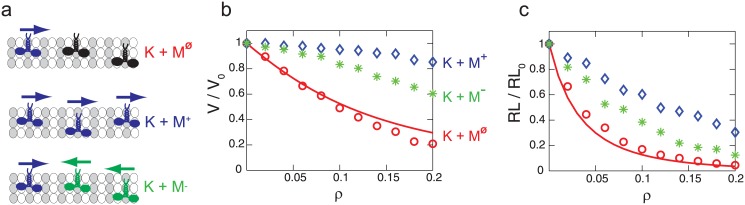
The velocity and run length of kinesins over the density of motors. (a) depicts the motions of the kinesin in (K + M^Φ^), (K + M^+^), and (K + M^−^) situations. Motors with an arrow directed to the right are moving kinesins. Motors with an arrow directed to the left are minus-end directed motors. Immobile kinesins are presented as motors without an arrow. *ρ* represents the molar ratio of immobile kinesins and tubulins for (K + M^Φ^), the molar ratio of walking kinesins and tubulins for (K + M^+^), or the molar ratio of minus-end directed motors and tubulins for (K + M^−^). Note that the MT is saturated with kinesins when *ρ* is about 0.43. The line denotes the velocity and run length for (K + M^Φ^) obtained from Eqs 5 and 6. The results shown as circles, stars and diamonds are calculated from our stochastic model.

Since the state of immobile kinesins is the same with the state of walking kinesins when they wait for ATP to bind, the interactions between moving kinesins and immobile kinesins, which is characterized in this study, can be used to consider the interaction between moving motor proteins. The minus-end directed motors, such as dyneins, are considered in our model simply by reversing the direction of the steps of kinesin. (K + M^Φ^) represents the situation when the motion of kinesin is disturbed by immobile kinesins. Also, kinesins can walk while surrounded by other kinesins (K + M^+^) or in the presence of minus-end directed motors (K + M^−^). These cases are depicted in Fig 9a. The changes of the kinesin motion by surrounding motors are related to the probability of the kinesin to interact with other motors. That probability is high for (K + M^Φ^), and is small for (K + M^+^) due to the motility of the surrounding motors. Thus, (K + M^+^) has the smallest reduction in velocity and run length, whereas K + M^Φ^ causes the most significant decreases in velocity and run length, as shown in Fig 9b and 9c.

### Length of NLs

The proposed model can be used to study the effects of the numbers of AAs at the NLs. This changes the probabilities of sideway and diagonal stepping and the interference with obstacles. These quantities are predicted by using our model. For example, Hoeprich et al. [46] predicted that the probability of stepping to each binding sites is slightly altered depending on the number of AAs in the NLs. Our model provides consistent results with their findings when the number of AAs in the NL is changed from 14 to 17, i.e., changed to the number of AAs in the NLs of kinesin-2. Our model predicts that when NLs of kinesin-2 are used, the probabilities of sideway and diagonal stepping increase by 1% compared to the same probabilities for kinesin-1.

Additionally, according to our model, when a single obstacle of *R*_obs_ = 5 nm is located in front of a kinesin (i.e., site (7)), the value of *A*^int^ is 0.46 ms/nm for kinesin-2, which is smaller than the respective one (i.e., 0.58 ms/nm) for kinesin-1. Hence, the interference between the free head of kinesin-2 and obstacles is not as strong as the interference between kinesin-1 and obstacles. This means that kinesin-2 is less likely to unbind from the MT in the presence of obstacles than kinesin-1. Therefore, the model predictions are consistent with the measurements of Hoeprich et al. [46] who observed that the run length of kinesin-1 decreased due to the presence of tau proteins, whereas and the run length of kinesin-2 did not decrease significantly.

### Probability of large step

**Fig 10:**
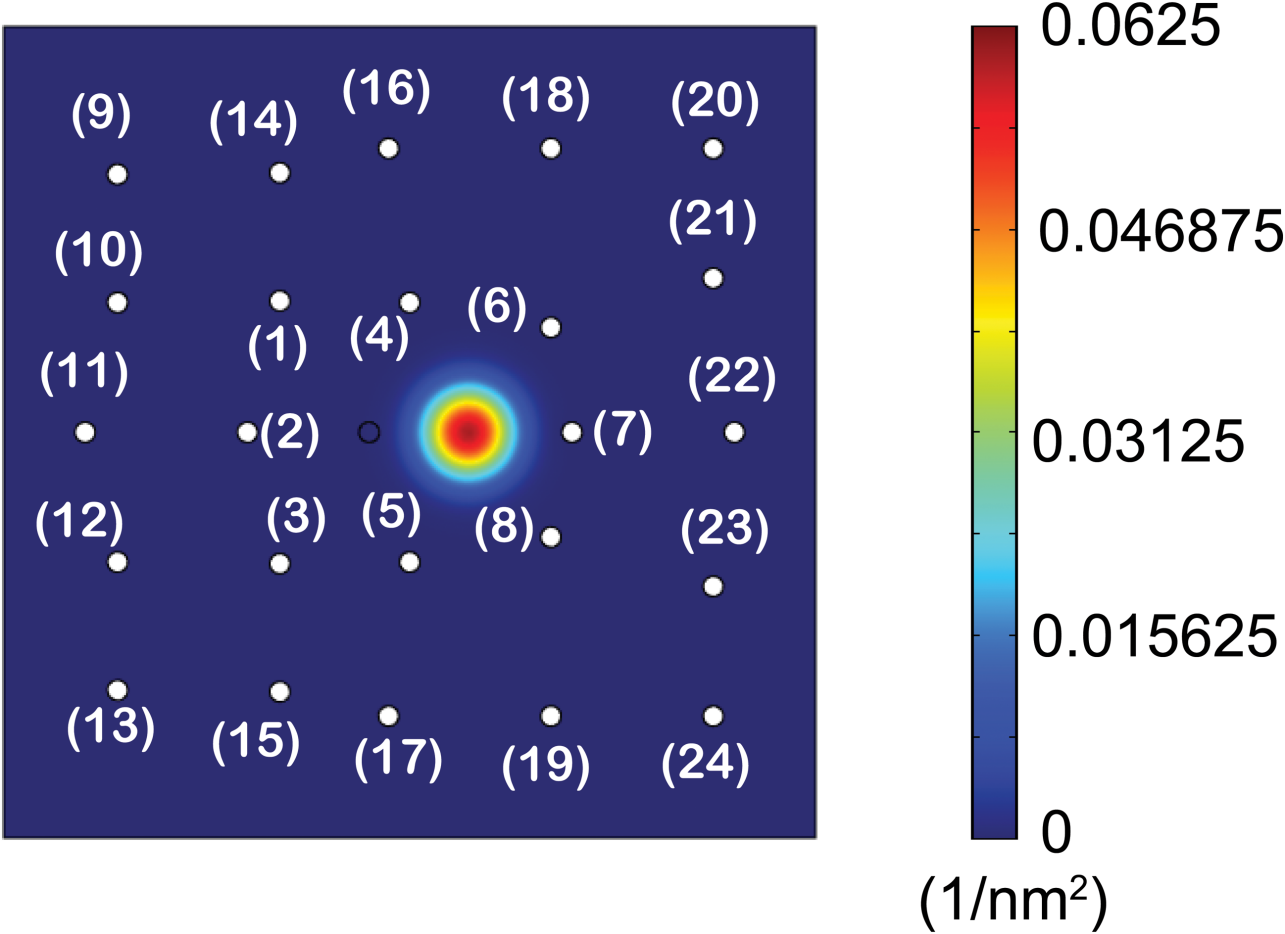
Spatial probability density of the position of the free head in the large domain.

Another use of our model includes studying calculating the probability of larger steps (i.e., probability of stepping to distant binding sites). The domain, which is shown in Fig 4a, is extended to consider *P*_*b*, *i*_ of twenty four neighboring binding sites. Also, the forces acting on the head (i.e., *F*_*x*_(*x*, *y*) and *F*_*y*_(*x*, *y*)) are calculated in this extended domain. Next, the diffusion of the free head is obtained, as shown in Fig 10. The result suggests that binding to sites far from the fixed head is possible but the probability of such events is very small. The sum of the probabilities (= *P*_*b*, 9_ + *P*_*b*, 10_ + *P*_*b*, 11_ + … + *P*_*b*, 24_) is about 1.3 × 10^−8^.

## Discussion

A diffusion model was developed in this study to predict two dimensional motion of kinesin which is necessary to bypass obstacles. Next, the interference between the walking kinesin and immobile kinesins was characterized by comparing the kinesin motion obtained from the diffusion model with experimentally measured velocities and run lengths. By using that interaction, the effects of other motor proteins on kinesins are predicted when they move on the same MT. If several kinesins share one MT, their velocities are almost not affected by other kinesins on the MT. The deceleration of kinesins by minus-end directed motors is also not considerable. These results suggest that cells can handle several intracellular transport processes effectively with a small number of MTs. However, both the velocity and run length decrease considerably in the presence of immobile kinesins. As a result, the malfunction of a few motors could degrade the whole transport system.

The diffusion model developed to capture the interaction between kinesins (or between kinesins and other motor proteins) can be also applied to other types of obstacles, such as tau proteins which are attached to the MT. The run length of kinesins has been observed to decrease for an excessive density of tau proteins in several experiments [18, 20, 47, 48]. Also, experiments have shown that the motion of single kinesins (with no cargo) is not slowed down by tau proteins. However, the transport velocity of cargoes can be modulated by tau proteins. Large cargoes such as mitochondria are transported by several kinesins to overcome large viscous forces in the cytoplasm. It is observed that the unbinding rate of kinesins increases and the rebinding rate of kinesins to the MTs decreases in the presence of tau proteins [18, 47–49]. Thus, the number of kinesins which effectively transport a large cargo is decreased by tau proteins, leading to slower transport of large cargo. Also, this decelerated cargo transport and the attached kinesins can behave as obstacles for other kinesin molecules. As a result, the entire transport system cannot operate properly.

At this point, it is worth to note that the effects of obstacles on the motion of kinesin is important because several neurodegenerative diseases such as Alzheimer’s, Parkinson’s, and Huntington’s disease have been reported to be related to an abnormal intracellular transport [50–52]. The negative effects of obstacles on the intracellular transport have been observed also *in vivo*. Specifically, the transport of various types of cargoes was decelerated or failed due to an excessive number of tau proteins [53–55]. Thus, the characterization of the motion of kinesin in the presence of tau proteins is necessary to reveal quantitative aspects of the relation between neurodegenerative diseases and tau proteins. Our diffusion model can be effectively used to obtain this kind of information and thus shed light on the relation between excessive tau proteins and degradation of axonal transport.

## Supporting Information

S1 File Deterministic and stochastic model for the motion of kinesin in the presence of obstacles. Fig A describes several situations where a kinesin molecule encounters obstacles. Table A presents the parameters to calculate velocity using the deterministic model. The values of parameters of the mechanistic model are presented in Table B.
(ZIP)
