# Supplementary material for: Effects of Obstacles on the Dynamics of Kinesins, Including Velocity and Run Length, Predicted by a Model of Two Dimensional Motion
Source: PLoS One. 2016 Jan 25;11(1):e0147676. doi: 10.1371/journal.pone.0147676 (PMC4726810; doi:10.1371/journal.pone.0147676)
Supplement: S1 File — Fig A describes several situations where a kinesin molecule encounters obstacles. Table A presents the parameters to calculate velocity using the deterministic model. The values of parameters of the mechanistic model are presented in Table B. (ZIP) [file pone.0147676.s001.zip › S1_text_r2.pdf]

Supporting Information

Deterministic model

The mean velocity and run length in the presence of static obstacles can be estimated by using the motion of kinesin when the obstacles are ahead of the kinesin (i.e., at sites (6), (7), and (8) in Fig. 3 (a1)). The obstacles at those sites have considerable effects on the kinesin motion because the free head diffuses mostly near the forward sites.

The velocity is determined by the average time T_d between steps of the kinesin [2] and the average moving distance d_{step} along the MT axis per step. d_{step} is equal to $8\frac{N_{\text{fw}}}{N_{\text{tot}}}$, where N_{tot} is the average of the total number of steps, and N_{fw} is the average of the number of steps to forward sites (i.e., sites (6), (7), and (8) in Fig. 3 (a1)). N_{tot} and N_{fw} are obtained before the kinesin unbinds from the MT. Thus, the velocity is calculated as

$$V = \frac{d_{\text{step}}}{T_d} = \frac{8\ N_{\text{fw}}}{N_{\text{tot}}}\ \frac{1}{T_d}. \tag{S1}$$

Because N_{fw} is almost equal to N_{tot} in the absence of obstacles, the velocity V_0 in the absence of obstacles can be calculated as $\frac{8}{T_d}$, where T_d is the sum of the time for the stepping motion and the time for the chemical reaction. Also, the time for the stepping motion is very short compared to the time for the chemical reaction. Therefore, the value of T_d in the presence of obstacles is similar to its value in the absence of obstacle. Thus, the ratio of the velocity in the presence and in the absence of obstacles can be obtained as $\frac{V(\rho)}{V_0} = \frac{1}{N_{\text{tot}}(\rho)/N_{\text{fw}}(\rho)}$. The denominator can be approximated as the average number of steps required to proceed 8 nm along the MT axis. Thus, the ratio of velocities can be obtained as

$$\frac{V(\rho)}{V_0} \simeq \frac{1}{\sum_{i=1}^{q} p_{\text{obs},i}(\rho)\ n_{\text{step},i} + \left(1 - \sum_{i=1}^{q} p_{\text{obs},i}(\rho)\right)}, \tag{S2}$$

where $p_{\text{obs},i}$ is the probability to encounter obstacles with a specific arrangement. Examples of $p_{\text{obs},i}$ are shown in Figure A (a). Note that the terms of i from 1 to m_{obs} correspond to a kinesin which confronts a single obstacle, and the terms of i from $m_{\text{obs}} + 1$ to $2m_{\text{obs}}$ correspond to a kinesin which confronts two series of obstacles. Table A shows the number of steps $n_{\text{step},i}$ corresponding to $p_{\text{obs},i}$.

m_{obs}	R_{obs}	$n_{\text{step},1}$	$n_{\text{step},2}$	$n_{\text{step},3}$
1	4	1.35	-	-
1	5	1.56	-	-
3, 5, or 9	4	2.01	3.04	2.01
3, 5, or 9	5	2.73	3.68	2.73

Table A. The parameters of the deterministic model.

The run length along the MT axis is $8N_{\text{fw}}$, which is equal to $8\frac{N_{\text{fw}}}{N_{\text{tot}}}N_{\text{tot}}$, and N_{tot} is the same as the inverse of the unbinding probability per step (i.e., $N_{\text{tot}} = \frac{1}{P_{ub}}$) [1]. Thus, the run length can be calculated as

$$RL = 8\frac{N_{\text{fw}}}{N_{\text{tot}}}N_{\text{tot}} = 8\frac{N_{\text{fw}}}{N_{\text{tot}}}\frac{1}{P_{ub}}. \tag{S3}$$

Again, N_{fw} is almost equal to N_{tot} in the absence of obstacles. Therefore, the ratio of the run lengths with an without obstacles can be obtained as

$$\begin{aligned}\frac{RL(\rho)}{RL_0} &\simeq \frac{N_{\text{fw}}(\rho)}{N_{\text{tot}}(\rho)}\frac{P^0_{\text{ub}}}{< P_{\text{ub}}(\rho) >} \\ &\simeq \frac{V(\rho)}{V_0}\frac{P^0_{\text{ub}}}{\sum_{i=1}^{3} p_{\text{ub},i}\ \widetilde{p}_{\text{obs},i} + P^0_{\text{ub}}(1 - \sum_{i=1}^{3} \widetilde{p}_{\text{obs},i})},\end{aligned} \tag{S4}$$

where $< P_{\mathrm{ub}}(\rho) >$ is the average unbinding probability per step in the presence of obstacles. Note that $< P_{\mathrm{ub}}(\rho) >$ is different from P_{ub} in Eq. (4). P_{ub} represents the unbinding probability of when obstacles are near the kinesin. $< P_{\mathrm{ub}}(\rho) >$ is the average unbinding probability which considers both the situation when obstacles are located near the kinesin and the other situation when there are no obstacles around the kinesin. $\widetilde{p}_{\mathrm{obs},i}$ is the probability that two or three sites in front of a kinesin are occupied by obstacles, as shown in Figure A (b). $\widetilde{p}_{\mathrm{obs},i}$ can be calculated as

$$\begin{aligned}
\widetilde{p}_{\mathrm{obs},1} &\simeq \frac{1}{3}\sum_{i=1}^{3} p_{\mathrm{obs},i}\ n_{\mathrm{step},i} + \frac{1}{6}\sum_{i=4}^{9} p_{\mathrm{obs},i}\ n_{\mathrm{step},i}, \\
\widetilde{p}_{\mathrm{obs},2} &\simeq \frac{1}{3}\sum_{i=1}^{3} p_{\mathrm{obs},i}\ n_{\mathrm{step},i} + \frac{2}{3}\sum_{i=4}^{9} p_{\mathrm{obs},i}\ n_{\mathrm{step},i}, \\
\widetilde{p}_{\mathrm{obs},3} &\simeq \widetilde{p}_{\mathrm{obs},1},
\end{aligned} \tag{S5}$$

where $\sum_{i=1}^{3} p_{\mathrm{obs},i}\ n_{\mathrm{step},i}$ is the probability that the kinesin interacts with a single obstacle. Thus, the probability that the binding sites (6) and (7) are occupied by single obstacles can be approximated as $\frac{1}{3}\sum_{i=1}^{3} p_{\mathrm{obs},i}\ n_{\mathrm{step},i}$. With the same method, the probability that the binding sites (6) and (7) are occupied by two series of obstacles is $\frac{1}{6}\sum_{i=4}^{9} p_{\mathrm{obs},i}\ n_{\mathrm{step},i}$. $p_{\mathrm{ub},1-3}$ are unbinding probabilities per step corresponding to $\widetilde{p}_{\mathrm{obs},1-3}$. The values of $p_{\mathrm{ub},1-3}$ can be calculated using Eq. (4).

The consideration on single obstacles is sufficient when fitting the deterministic model to the experimental data for cases where the density of obstacles in the experiments is small. If the density of obstacles increases, the kinesin is also likely to confront two or more series of obstacles. Thus, the effects of single obstacle and two series of obstacles are incorporated into the model to calculate the velocity and run length, which are shown in Fig. 6. $n_{\mathrm{step},4-6}$ for immobile kinesins are obtained as 4.16, 8.43, and 10.1 from the diffusion model. By symmetry, $n_{\mathrm{step},7-9}$ have the same values as $n_{\mathrm{step},6-4}$. With the values of $n_{\mathrm{step},4-9}$, the values of the coefficients in Eqs. (5) and (5) are obtained as $a_1 = 7.04$, $a_2 = 25.29$, $b_1 = 24.28$, and $b_2 = 60.04$ by using Eqs. (S3) and (S5).

Stochastic model

To capture the stochastic motion of kinesins, the diffusion model is integrated with a previous mechanistic model [2]. The probability of the direction of the step, and the increase in the unbinding probability by obstacles are calculated by using the diffusion model. The mechanochemical cycle and the unbinding probability of kinesin are based on the previous model [2]. The several dynamics of kinesins (state transition in the mechanochemical cycle, unbinding from the MT, and direction of step) are stochastic processes. Thus, they are determined by comparing the calculated probabilities with a random number. To perform Monte-Carlo simulations, new random numbers are generated at every step.

The previous stochastic model did not consider side steps. Thus, the parameters of the previous model are slightly updated. The parameter values used in this study are presented in Table B.

The stochastic model can be used to obtain values for α_{obs}. In previous experiments which studied the effect of immobile kinesins on the walking kinesins, the displacement of kinesin was captured at every 0.1 s, and the spatial resolution was 40 nm. Thus, these values of time and spatial resolution are used with our stochastic model when calculating α_{obs}.

Parameter	Value	Unit
k_{1f}	2.83	$\mu M^{-1}s^{-1}$
$k_{1b,0}$	26.5	s^{-1}
k_{2f}	108.7	s^{-1}
Φ_c	1.295	nm
κ	4.837	pN/nm
$k_{D0,0}$	0.0285	s^{-1}
$P_{D1,0}$	0.00934	probability
d_0	0.043	nm
d_1	0.851	nm

Table B. The values of parameters of the mechanistic model.

Figures Legends

Figure A. Kinesin with obstacles characterized by $m_{\text{obs}} = 3$. (a) The probability $p_{\text{obs},i}$ that a kinesin encounters obstacles is equal to ρ. (a1)-(a3) There are several situations where a kinesin confronts a single obstacle. The corresponding probabilities $p_{\text{obs},1-3}$ are provided. (a4)-(a6) There are situations where a kinesin encounters two adjacent of obstacles. The corresponding probabilities $p_{\text{obs},4-6}$ are also provided. (b) The probability $\widetilde{p}_{\text{obs},i}$ that a kinesin interacts with obstacles with a specific arrangement can be calculated. Several cases are shown.

References

1. Telley IA, Bieling P, Surrey T. Obstacles on the microtubule reduce the processivity of Kinesin-1 in a minimal in vitro system and in cell extract. Biophys J. 2009;96(8):3341–3353.
2. Nam W, Epureanu BI. Highly loaded behavior of kinesins increases the robustness of transport under high resisting Loads. PLoS Comput Biol. 2015;11(3):e1003981–e1003981.
